# Genetic prion disease–related mutation E196K displays a novel amyloid fibril structure revealed by cryo-EM

**DOI:** 10.1126/sciadv.abg9676

**Published:** 2021-09-08

**Authors:** Li-Qiang Wang, Kun Zhao, Han-Ye Yuan, Xiang-Ning Li, Hai-Bin Dang, Yeyang Ma, Qiang Wang, Chen Wang, Yunpeng Sun, Jie Chen, Dan Li, Delin Zhang, Ping Yin, Cong Liu, Yi Liang

**Affiliations:** 1 Hubei Key Laboratory of Cell Homeostasis, College of Life Sciences, Wuhan University, Wuhan 430072, China.; 2 Interdisciplinary Research Center on Biology and Chemistry, Shanghai Institute of Organic Chemistry, Chinese Academy of Sciences, Shanghai 201210, China.; 3 University of Chinese Academy of Sciences, Beijing 100049, China.; 4 National Key Laboratory of Crop Genetic Improvement and National Centre of Plant Gene Research, Huazhong Agricultural University, Wuhan 430070, China.; 5 Department of Biophysics and Department of Pathology of Sir Run Run Shaw Hospital, Zhejiang University School of Medicine, Hangzhou, Zhejiang 310058, China.; 6 Key Laboratory for the Genetics of Developmental and Neuropsychiatric Disorders, Ministry of Education, Bio-X Institutes, Shanghai Jiao Tong University, Shanghai 200030, China.

## Abstract

Prion diseases are caused by the conformational conversion of prion protein (PrP). Forty-two different mutations were identified in human PrP, leading to genetic prion diseases with distinct clinical syndromes. Here, we report the cryo–electron microscopy structure of an amyloid fibril formed by full-length human PrP with E196K mutation, a genetic Creutzfeldt-Jakob disease–related mutation. This mutation disrupts key interactions in the wild-type PrP fibril, forming an amyloid fibril with a conformation distinct from the wild-type PrP fibril and hamster brain–derived prion fibril. The E196K fibril consists of two protofibrils. Each subunit forms five β strands stabilized by a disulfide bond and an unusual hydrophilic cavity stabilized by a salt bridge. Four pairs of amino acids from opposing subunits form four salt bridges to stabilize the zigzag interface of the two protofibrils. Our results provide structural evidences of the diverse prion strains and highlight the importance of familial mutations in inducing different strains.

## Teaser

The cryo-EM structure of genetic CJD mutation E196K fibril reveals a novel fibril polymorph of human prion protein.

## INTRODUCTION

Prions, which mean “protein infectious agents” and convert between structurally and functionally distinct states, were originally isolated and described by S. B. Prusiner (*1*–*3*). Prion diseases are infectious, fatal neurodegenerative diseases primarily caused by the conformational conversion of prion protein (PrP) from its cellular form (PrP^C^) into a protease-resistant, aggregated form (PrP^Sc^) in humans, cattle, sheep, and cervid species (*1*–*12*). Forty-two different mutations in the PrP gene (*PRNP*) were identified to cause a variety of genetic prion diseases, including genetic Creutzfeldt-Jakob disease (CJD), Gerstmann-Sträussler-Scheinker disease, and fatal familial insomnia (*2*, *4*, *6*, *10*). These disease-related mutations can form different strains with distinct conformations and display distinct clinical syndromes with different incubation times (*13*–*20*). Most of the mutations do not cause large conformational changes in PrP^C^, whose structure features a folded C-terminal globular domain containing three α helices and two very short antiparallel β sheets accompanied by a largely disordered N-terminal tail (*5*, *10*, *21*). Instead, the mutations were reported to induce spontaneous generation of PrP^Sc^ in the brain of patients with genetic prion diseases (*10*) and in the brain of transgenic mouse models with the mutations (*22*, *23*). Since prions were found in 1982 (*1*), great efforts have been dedicated to unravel the mysteries of the atomic structure of prion (*7*, *8*, *11*, *20*, *24*–*34*) and prion strains (*13*–*20*, *34*). Recently, we reported a cryo–electron microscopy (cryo-EM) structure of the amyloid fibril formed by full-length wild-type human PrP featuring a parallel in-register intermolecular β sheet architecture (*33*), which provides structural insights into the conversion from α helix–dominant PrP^C^ to β sheet–rich PrP^Sc^. In addition, Kraus and co-workers reported a cryo-EM structure of a hamster brain–derived prion (263K prion) fibril featuring a hydrophobic “Greek key” motif, a middle β arch, and a disulfide β arch (*34*). While that work (*34*) is still in preprint form, it provides a richness of information directly relevant to prion strains and structural diversity, stability, and glycosylation of PrP fibrils.

The first reported patient with E196K mutation in the *PRNP* gene, a genetic CJD-related mutation, was a 69-year-old French woman, who died 1 year after the first recorded symptoms (*35*). The patients carrying this mutation have an average age at onset of 71.2 ± 5.9 years old (*n* = 15) (*36*, *37*). As we note, there are more than three dozen familial mutations in PrP; most of them likely have a structural impact (*2*, *4*, *6*, *10*). In this study, we focus specifically on E196K mutation because of the following reasons. First, the patients carrying E196K mutation feature rapid disease progression and short disease duration (6.6 ± 3.5 months) (*36*, *37*), but the mechanism behind this phenomenon is unknown. Previous studies have shown that E196K mutation decreases the thermal stability of PrP^C^ and thus increases the propensity for PrP amyloid formation (*38*). Second, the atomic structure of wild-type PrP fibrils has shown that several familial mutations including K194E, E196K, and E211Q may break salt bridges essential for forming the protofilament interface in wild-type PrP fibrils and thus disrupt PrP fibril structure (*33*), but whether genetic prion disease–related mutation E196K displays a new amyloid fibril structure is unknown.

Here, we prepared homogeneous amyloid fibrils in vitro from recombinant, full-length human E196K PrP^C^ and determined the atomic structure by using cryo-EM. While the infectivity of these fibrils remains to be established, the structural features provide insights into the mechanism how familial mutations drive formation of different prion strains.

## RESULTS

### E196K forms amyloid fibrils morphologically distinct from wild-type PrP

We produced amyloid fibrils from recombinant, full-length human E196K PrP^C^ (residues 23 to 231) overexpressed in *Escherichia coli*, by incubating the purified protein in 20 mM tris-HCl buffer (pH 7.4) containing 2 M guanidine hydrochloride and shaking at 37°C for 7 to 9 hours. E196K fibrils were dialyzed against NaAc buffer, purified by ultracentrifugation, resuspended in NaAc buffer, and examined by transmission electron microscopy (TEM) without further treatment.

**Fig. 1.**
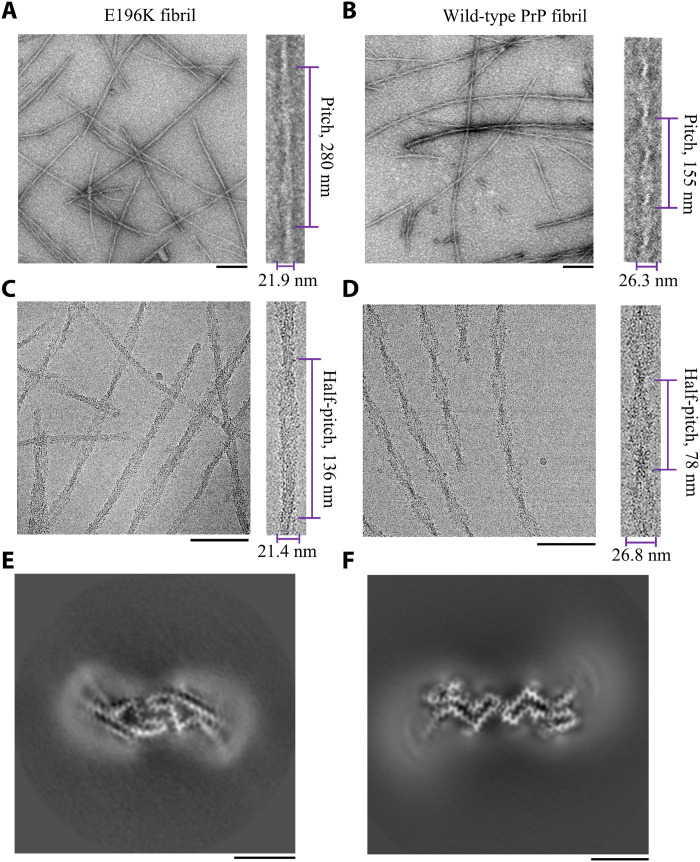
Comparison of the images of the E196K fibril and the wild-type fibril. (**A** and **B**) Negative-staining TEM images of amyloid fibrils from full-length wild-type human PrP (B) and its E196K variant (A). Enlarged section of (A) (right) or (B) (right) showing two protofibrils intertwined into a left-handed helix, with a fibril full width of 21.9 ± 0.7 nm (*n* = 8) (A) or 26.3 ± 0.9 nm (*n* = 8) (B) and a helical pitch of 280 ± 7 nm (*n* = 8) (A) or 155 ± 4 nm (*n* = 8) (B). The scale bars represent 200 nm. (**C** and **D**) Raw cryo-EM images of amyloid fibrils from E196K (C) and its wild-type form (D). Enlarged section of (C) (right) or (D) (right) showing two protofibrils intertwined into a left-handed helix, with a fibril full width of 21.4 ± 0.7 nm (*n* = 8) (C) or 26.8 ± 1.2 nm (*n* = 8) (D) and a half-helical pitch of 136 ± 6 nm (*n* = 8) (C) or 78 ± 4 nm (*n* = 8) (D). The scale bars represent 100 nm. E196K formed amyloid fibril with a morphology distinct from the one formed by wild-type PrP. The helical pitch, half-helical pitch, and width were measured and expressed as the means ± SD of values obtained in eight independent measurements. (**E** and **F**) Cross-sectional view of the 3D map of the E196K fibril (E) showing two protofibrils also forming a dimer but with a conformation distinct from the wild-type fibril (F). Scale bars, 5 nm.

Negative-staining TEM imaging showed that E196K PrP^C^ formed homogeneous and unbranched fibrils (Fig. 1A), similar to wild-type PrP fibrils formed at the same conditions (Fig. 1B). The E196K fibril is composed of two protofibrils intertwined into a left-handed helix, with a fibril full width of 21.9 ± 0.7 nm and a helical pitch of 280 ± 7 nm (Fig. 1A). By comparison, the wild-type fibril is also composed of two protofibrils intertwined into a left-handed helix, but with a fibril full width of 26.3 ± 0.9 nm and a short helical pitch of 155 ± 4 nm (Fig. 1B). Thus, E196K formed an amyloid fibril with morphology distinct from wild-type PrP.

Congo red binding assays showed a red shift of the maximum absorbance, from 490 to 550 nm, in the presence of E196K fibrils (fig. S1A), which is typical of amyloid fibrils (*33*). This is similar to wild-type PrP fibrils formed at the same conditions (*33*).

Proteinase K digestion of the E196K fibrils generated a predominant band with an apparent molecular weight of 14 to 15 kDa (fig. S1C). This is different from wild-type PrP fibrils formed at the same conditions, which generated a predominant band with an apparent molecular weight of 15 to 16 kDa (*33*, *39*, *40*). Furthermore, at the same protease:PrP molar ratios, the optical density of the 14- to 15-kDa band in the E196K fibrils was remarkably weaker than that of the 15- to 16-kDa band in the wild-type fibrils (fig. S1, B and C). Bocharova and co-workers reported that proteinase K digestion of full-length wild-type mouse PrP fibrils generated a band with an apparent molecular weight of 21 kDa (*41*), but we and others (*40*) did not observe a 21-kDa band after proteinase K digestion of full-length wild-type human PrP fibrils (fig. S1B) and the E196K fibrils (fig. S1C), possibly because amyloid fibrils formed by PrPs from different mammalian species have remarkably different proteinase K–resistant features (*39*). In addition, we observed two other bands with apparent molecular weights of 12 and 10 kDa after proteinase K digestion of the E196K fibrils (fig. S1C), which is similar to wild-type PrP fibrils (fig. S1B). Together, the data showed that the E196K fibril exhibits a distinct morphology with weaker protease resistance activity compared to the wild-type fibril.

### Cryo-EM structure of E196K fibrils

We determined the atomic structure of the E196K amyloid fibrils by cryo-EM (Table 1). The cryo-EM micrographs and two-dimensional (2D) class average images show that the E196K fibril is composed of two protofibrils intertwined into a left-handed helix (Fig. 1C and fig. S2A), with a helical pitch longer than the wild-type fibril (Fig. 1, C and D). Furthermore, two protofilaments in each E196K fibril is arranged in a staggered manner (fig. S2, B and C), similar to wild-type PrP fibrils formed at the same conditions (*33*). The fibrils are morphologically homogeneous, showing a fibril full width of 21.4 ± 0.7 nm (Fig. 1C and fig. S2A). This is narrower than wild-type PrP fibrils (*33*) but similar in size to previously described ex vivo, infectious PrP^Sc^ fibrils, which showed a width of ~20 nm based on negative staining on TEM (*31*, *42*).

**Table 1.** Cryo-EM data collection, refinement, and validation statistics.

	**E196K fibril (EMD-30887, PDB 7DWV)**
**Data collection and processing**	
Magnification	29,000
Voltage (kV)	300
Camera	K2 summit (Titan Krios)
Frame exposure time (s)	0.2
Movie frames (*n*)	40
Electron exposure (e^−^/Å^2^)	64
Defocus range (μm)	−3.0 to −1.5
Pixel size (Å)	1.014
Symmetry imposed	*C*1
Box size (pixel)	400
Interbox distance (Å)	40.6
Micrographs collected (*n*)	4883
Segments extracted (*n*)	619,926
Segments after Class2D (*n*)	560,963
Segments after Class3D (*n*)	24,083
Map resolution (Å)	3.07
FSC threshold	0.143
Map resolution range (Å)	2.20–4.01
**Refinement**	
Initial model used	6LNI
Model resolution (Å)	3.07
FSC threshold	0.143
Model resolution range (Å)	3.07
Map sharpening *B* factor (Å^2^)	−43.26
Model composition	
Nonhydrogen atoms	2064
Protein residues	258
Ligands	0
*B* factors (Å^2^)	
Protein	101.56
R.m.s. deviations	
Bond lengths (Å)	0.007
Bond angles (°)	0.795
**Validation**	
MolProbity score	2.54
Clashscore	16.67
Poor rotamers (%)	0
Ramachandran plot	
Favored (%)	73.17
Allowed (%)	26.83
Disallowed (%)	0

**Fig. 2.**
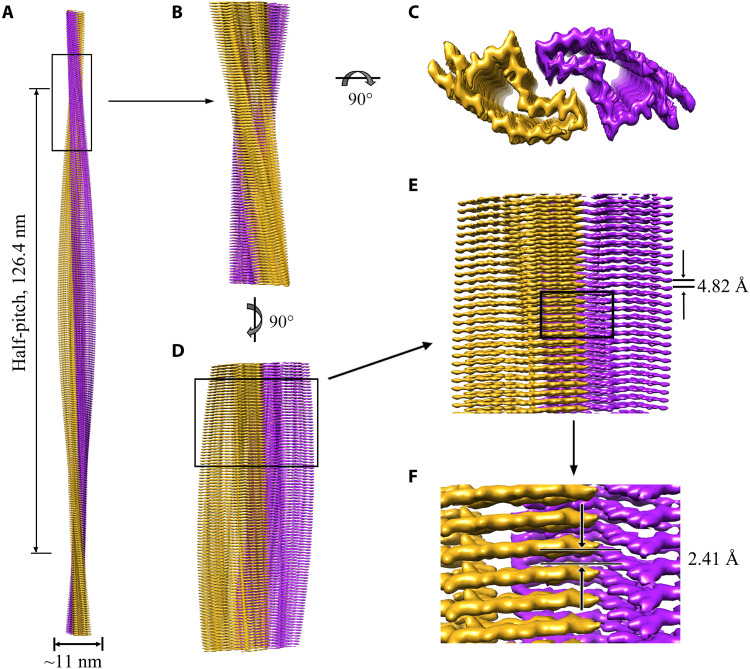
Cryo-EM structure of E196K fibrils. (**A**) 3D map showing two protofibrils intertwined into a left-handed helix, with a fibril core width of ~11 nm and a half-helical pitch of 126.4 nm. The two intertwined protofibrils are colored purple and gold, respectively. (**B**) Enlarged section showing a side view of the density map. (**C**) Top view of the density map. (**D**) Another side view of the density map after 90° rotation of (B) along the fibril axis. (**E**) Close-up view of the density map in (D) showing that the subunits in each protofibril stack along the fibril axis with a helical rise of 4.82 Å. (**F**) Close-up view of the density map in (E) showing that the subunits in two protofibrils stack along the fibril axis with a helical rise of 2.41 Å.

Using helical reconstruction in RELION3.1 (*43*), we determined a density map of the ordered core of E196K fibrils, with an overall resolution of 3.07 Å, which features well-resolved side-chain densities and clearly separated β strands along the helical axis (Fig. 1E and fig. S3). The 3D map showed two protofibrils in the E196K fibril intertwined into a left-handed helix, with a half-helical pitch of 126.4 nm (Fig. 2A), which is remarkably longer than that of the wild-type fibril (*33*). The width of the fibril core is ~11 nm (Fig. 2A). This is narrower than wild-type PrP fibrils formed at the same conditions (*33*) but similar in size to previously described ex vivo, infectious PrP^Sc^ fibrils, which showed a width of ~10 nm based on cryo-EM images (*28*). The wild-type fibril full width in Fig. 1 (B and D) was 26.3 ± 0.9 and 26.8 ± 1.2 nm, respectively. This is remarkably wider than the wild-type fibril core width of ~14 nm (*33*), possibly because residues 23 to 169 for the wild-type fibril were not modeled in the cryo-EM density (*33*). Cross-sectional view of the 3D map of the E196K fibril (Fig. 1E) shows two protofibrils conformationally distinct from those of the wild-type PrP fibril (Fig. 1F). The protofilaments in the E196K fibril form a dimer with a screw symmetry of approximately 2_1_ (Fig. 2, B to D). The subunits in each E196K protofibril stack along the fibril axis with a helical rise of 4.82 Å and twist of −0.68° (Fig. 2E), and the subunits in two protofibrils stack along the fibril axis with a helical rise of 2.41 Å (Fig. 2F).

**Fig. 3.**
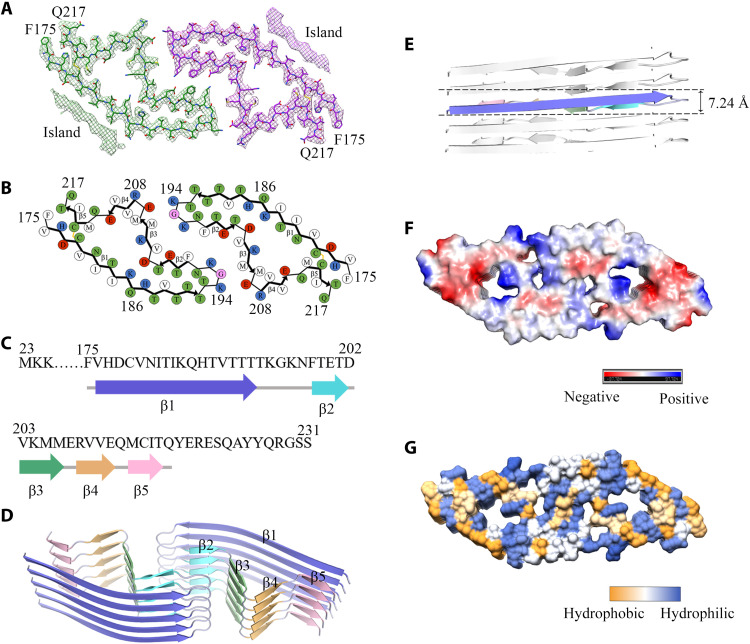
Atomic structure of E196K fibrils. (**A**) Cryo-EM map of E196K fibrils with the atomic model overlaid. Two identical densities (termed two islands) flanking the two protofibrils, which are colored green and purple, respectively. Each island is located on the opposing side of hydrophobic side chains of Val^180^, Ile^182^, and Ile^184^ in each monomer. (**B**) Schematic view of the E196K fibril core. Residues are colored as follows: white, hydrophobic; green, polar; red and blue, negatively and positively charged, respectively; and magenta, glycine. β strands are indicated with bold lines. E196K fibrils are stabilized by a disulfide bond (yellow line) formed between Cys^179^ and Cys^214^ in each monomer, also visible in (A). (**C**) Sequence of the fibril core comprising residues 175 to 217 from E196K, with the observed five β strands colored blue (β1), cyan (β2), green (β3), orange (β4), and pink (β5). The dotted line corresponds to residues 23 to 174 not modeled in the cryo-EM density. (**D**) Ribbon representation of the structure of an E196K fibril core containing five molecular layers and a dimer. (**E**) As in (D), but viewed perpendicular to the helical axis, revealing that the height of one layer of the subunit along the helical axis is 7.24 Å. (**F**) Electrostatic surface representation of the structure of an E196K fibril core containing five molecular layers and a dimer. (**G**) Hydrophobic surface representation of the structure of an E196K fibril core as in (D). (F and G) Two pairs of amino acids (Lys^194^ and Glu^207^; Lys^196^ and Glu^200^) from opposing subunits form four salt bridges at the zigzag interface of the two protofibrils. The surface of two opposing subunits is shown according to the electrostatic properties (F) or the hydrophobicity (G) of the residues.

We unambiguously built an E196K fibril model comprising residues 175 to 217 at 3.07 Å (Fig. 3) and an unmasked E196K fibril model comprising residues 171 to 222 at 3.59 Å (fig. S4), both of which are slightly shorter than the wild-type PrP fibril core comprising residues 170 to 229 (*33*). Side-chain densities for most residues in the E196K fibril core had high local resolution (2.20 to 3.28 Å) (Fig. 3, A and B, and fig. S4, A to C). The exterior of the E196K fibril core is mostly hydrophilic. The fibril core is stabilized by an intramolecular disulfide bond between Cys^179^ and Cys^214^ (Fig. 3, A and B, and figs. S4, B and C, and S5, A and B) (also present in both PrP^C^ and PrP^Sc^) (*24*, *27*, *28*, *30*) and a hydrogen bond between His^177^ and Thr^216^ (fig. S5, A and B) in each monomer. In sharp comparison to the highly hydrophobic cavity observed in the wild-type fibril, the E196K fibril core is composed of an unusual hydrophilic cavity in each subunit (Fig. 3, B and G, and fig. S4C), which may decrease the fibril stability of E196K. Lys^185^ and Asp^202^ from the same subunit form a salt bridge, and His^187^ and Asp^202^ from the adjacent subunit form another salt bridge to stabilize the hydrophilic cavity (fig. S6, A to C).

We observed two unidentified densities flanking the two protofibrils in the E196K fibril, termed two islands (Fig. 3A and fig. S4), which are reminiscent of those islands observed in the structures of filaments formed by α-synuclein hereditary disease mutant H50Q (*44*). Each island, probably comprising residues 136 to 142 from E196K and forming a β strand (β0), is located on the opposing side of hydrophobic side chains of Val^180^, Ile^182^, and Ile^184^ in each monomer (fig. S4, B to D). The side chains of Val^180^, Ile^182^, Ile^184^, Pro^137^, Ile^139^, and Phe^141^ form a hydrophobic steric zipper-like interface, thereby stabilizing the E196K fibrils. The presence of two islands represents one of the major structural differences between E196K and wild-type fibrils.

Five β strands (β1 to β5) and six β strands (β0 to β5) are present in the E196K fibril core structure at 3.07 Å (Fig. 3, B to D) and an unmasked structure at 3.59 Å (fig. S4C), respectively. The E196K fibril core features a compact fold containing a long β strand (β1) and four short β strands (β2 to β5). This is different from a previously observed wild-type PrP fibril core, which contains six short β strands (β1 to β6) (*33*). The height of one layer of the subunit along the helical axis is 7.24 Å (Fig. 3E). A U-turn between β1 and β2 containing residues ^193^TKGKN^197^ enables antiparallel cross-β packing of the first part of β1 against β2 and a disulfide bond between Cys^179^ in β1, and Cys^214^ in β5 enables antiparallel cross-β packing of the last part of β1 against β5 (Fig. 3, B and D, and fig. S4C).

In the E196K fibril, two pairs of amino acids (Lys^194^ and Glu^207^; Lys^196^ and Glu^200^) from opposing subunits form four salt bridges at the zigzag interface of the two protofibrils (Figs. 2C and 3, A, B, F, and G, and fig. S4, B and C). The interfaces in the E196K fibril feature mixed compositions of hydrophilic and hydrophobic side chains (Fig. 3, B and G, and fig. S4C). They are reminiscent of those interfaces observed in the structures of Tau filaments extracted from the brains of patients with corticobasal degeneration (*45*) and amyloid fibrils formed by the S20G mutation in human amylin (*46*). In the wild-type PrP fibril, however, Lys^194^ and Glu^196^ from opposing subunits form two salt bridges that create a hydrophilic cavity at the interface of the two protofibrils with two additional unidentified densities (*33*).

**Fig. 4.**
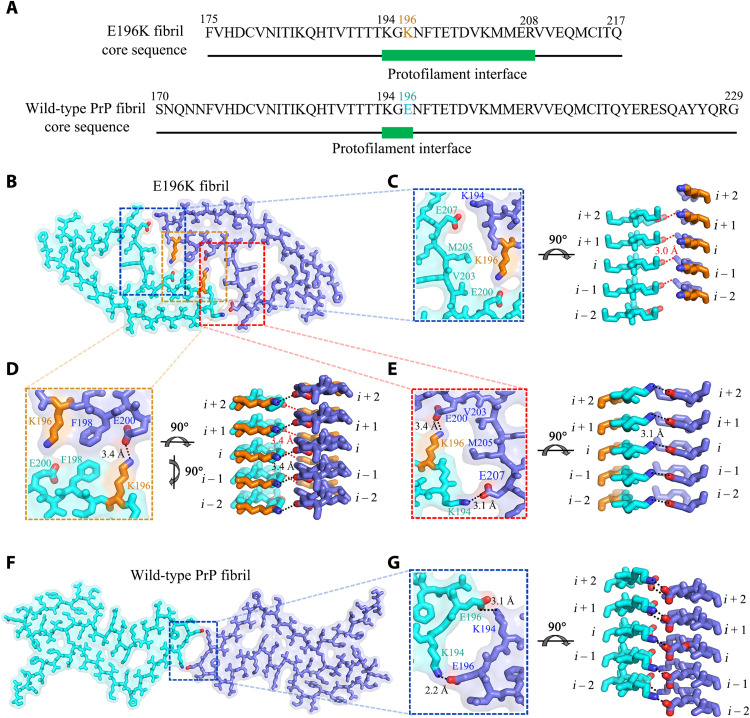
Comparison of protofilament interfaces of E196K and wild-type fibrils. (**A**) The primary sequences of the E196K fibril core and the wild-type fibril core. The green bar marks the region of the protofilament interface. Lys^196^ in E196K variant and Glu^196^ in wild-type PrP are highlighted in orange and cyan, respectively. (**B** and **F**) Two space-filled models overlaid onto stick representations of the E196K fibril (B) and the wild-type PrP fibril (PDB 6LNI) (*33*) (F), respectively, in which one protofibril is shown in cyan and another in blue. Lys/Glu pairs that form salt bridges are highlighted in red (oxygen atoms in Glu), blue (nitrogen atom in Lys), and orange (Lys^196^), and the dimer interface is magnified in (C) to (E) and (G). (**C** to **E**) Magnified top views of the three regions of the zigzag interface between E196K protofibrils, where four pairs of amino acids (Glu^207^ and Lys^194^; Glu^200^ and Lys^196^; Lys^196^ and Glu^200^; and Lys^194^ and Glu^207^) from opposing subunits form four salt bridges. Two side views (right) highlighting a strong salt bridge between Glu^207^ (*i*) and Lys^194^ or between Glu^200^ (*i*) and Lys^196^ from its opposing adjacent subunit (*i* − 1), with a distance of 3.0 or 3.4 Å (red). Two side views (right) highlighting a strong salt bridge between Lys^196^ (*i*) and Glu^200^ or between Lys^194^ (*i*) and Glu^207^ from its opposing subunit (*i*), with a distance of 3.4 or 3.1 Å (black). (**G**) A magnified top view of the dimer interface between wild-type PrP protofibrils, where two pairs of amino acids (Lys^194^ and Glu^196^; Glu^196^ and Lys^194^) from opposing subunits form two salt bridges. A side view (right) highlighting a strong salt bridge between Lys^194^ (*i*) and Glu^196^ or between Glu^196^ (*i*) and Lys^194^ from its opposing subunit (*i*), with a distance of 2.2 or 3.1 Å (black).

**Fig. 5.**
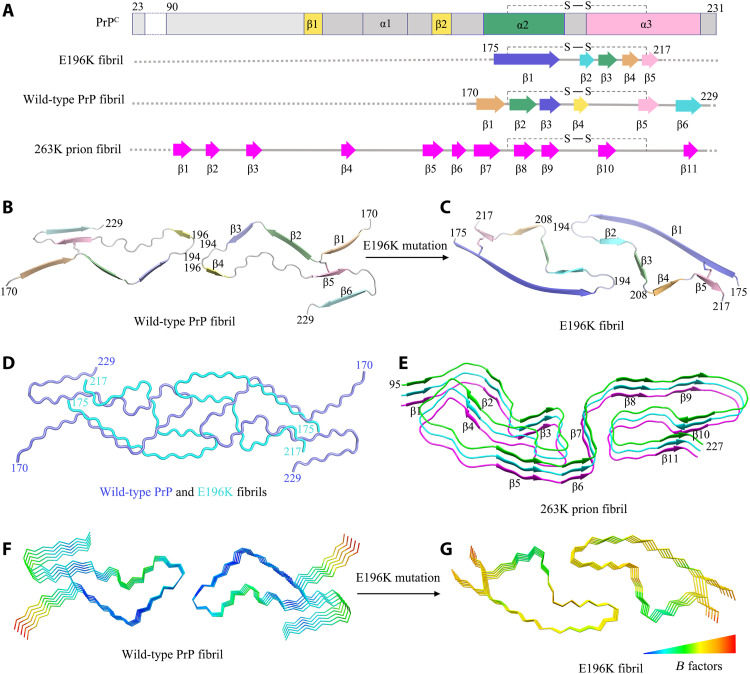
Comparison of the structures of PrP^C^, the E196K fibril, the wild-type PrP fibril, and the 263K prion fibril. (**A**) Sequence alignment of the full-length wild-type human PrP^C^ (23 to 231) monomer (PDB 1QLX) (*21*), the E196K fibril core comprising residues 175 to 217 from E196K with the observed five β sheets and one disulfide bond between Cys^179^ in β1 and Cys^214^ in β5, the wild-type PrP fibril core comprising residues 170 to 229 from wild-type PrP (PDB 6LNI) (*33*), and a hamster brain–derived prion (263K prion) fibril core comprising residues 95 to 227 with the observed 11 β sheets (*34*). S─S denotes a single disulfide bond. Dotted line (A) corresponds to residues 23 to 174 for the E196K fibril, residues 23 to 169 for the wild-type fibril, and residues 23 to 94 for the 263K prion fibril, respectively, which were not modeled in the cryo-EM density. (**B** and **C**) Ribbon representation of the structures of a wild-type PrP fibril core (PDB 6LNI) (*33*) (B) and an E196K PrP fibril core (C), both of which contain one molecular layer and a dimer with a single disulfide bond in each monomer. (**D**) Overlay of the structures of a wild-type PrP fibril core (blue) (PDB 6LNI) (*33*) and an E196K PrP fibril core (cyan). (**E**) Ribbon representation of the structure of a 263K prion fibril core containing three molecular layers and a monomer (*34*). (**F** and **G**) *B* factor representation of the structures of a wild-type PrP fibril core (PDB 6LNI) (*33*) (F) and an E196K PrP fibril core (G), both of which contain five molecular layers and a dimer. The C^α^ atoms of the fibrils are colored according to *B* factors, ranging from blue (69 Å^2^, lowest) to red (121 Å^2^, highest). The local region comprising residues 180 to 194 has remarkably higher flexibility in the E196K fibril (yellow) (G) compared to that in the wild-type fibril (blue) (F).

We then compare protofilament interfaces of E196K and wild-type fibrils in detail (Fig. 4). The E196K fibril has a long interface comprising residues 194 to 208, but the wild-type fibril features a very short interface comprising only three residues 194 to 196 (Fig. 4A). Four pairs of strong intermolecular salt bridges formed by Glu^207^ and Lys^194^ and Glu^200^ and Lys^196^ (with distances less than 4 Å; Fig. 4, B to E) and two pairs of weak salt bridges formed by Glu^207^ and Arg^208^ from the same subunit (with a distance of 6.3 Å; Figs. 3B and 4, B to E) are identified in the zigzag interface between E196K protofibrils. In contrast, two strong intermolecular salt bridges between Lys^194^ and Glu^196^ are formed at the dimer interface between wild-type PrP protofibrils (with distances less than 4 Å; Fig. 4, F and G). Thus, the E196K mutation disrupts the key salt bridges in wild-type PrP fibrils and results in a rearrangement of the overall structure, forming an amyloid fibril with a conformation distinct from the wild-type fibril (Figs. 4 and 5 and fig. S5).

### The E196K mutation significantly decreases the conformational stability of PrP fibrils

Compared to the wild-type fibril, the E196K fibril features a smaller and distinct fibril core with an unusual hydrophilic cavity in the center. We next examined whether the E196K fibril exhibits distinct conformational stability from the wild-type fibril. Chemical and/or thermal denaturation was widely used to evaluate the conformational stability of proteinase-resistant PrP fibrils (*19*, *47*, *48*). A strong chaotropic salt, guanidine thiocyanate, was used in our denaturation assay (fig. S7A). The *C*_1/2_ value of the wild-type PrP fibril is 2.00 ± 0.03 M (fig. S7B), which is consistent with previous data (*47*). Notably, the *C*_1/2_ value of the E196K fibril is 1.54 ± 0.06 M (fig. S7B), which is significantly lower than the wild-type fibril, suggesting that the E196K fibril is less stable than the wild type. The lability of E196K fibrils was confirmed by our salt gradient experiments (fig. S8A). Intriguingly, the *C*_1/2_ value of the E196K fibril in the presence of 150 mM or 1 M NaCl is 1.53 ± 0.06 M (fig. S8B) or 1.77 ± 0.10 M (fig. S8C), which is significantly lower than that of the wild-type fibril in the presence of 150 mM (2.00 ± 0.05 M; fig. S8B) or 1 M salt (2.47 ± 0.13 M; fig. S8C), suggesting that the E196K fibril is less stable than the wild type at physiological ionic strength and high salt concentration.

To validate the stability differences between E196K and wild-type fibrils, we further measured their thermostabilities. The PrP fibrils were incubated with 6% SDS under a thermal gradient from 25 to 100°C, and the soluble PrP disassembled from fibrils was measured by SDS–polyacrylamide gel electrophoresis (SDS-PAGE) (fig. S7, C and D). To determine the melting temperature (*T*_m_), we normalized the amount of soluble PrP monomers as a ratio of the density of soluble PrP monomer band at each temperature over the density of soluble PrP monomer band at 100°C and presented thermal stability data in the form of a plot (normalized amount of soluble PrP monomers versus temperature) (fig. S7E). The results showed that the *T*_m_ value of E196K fibrils is ~75°C, substantially lower than that of the wild-type fibrils (~95°C) (fig. S7E). Together, these results demonstrate that the E196K fibril has a significantly lower conformational stability compared to the wild-type fibril.

However, it is not fully obvious why the structure of the sequence variant would induce greater fibril lability. While E196K fibrils exhibit a presumably hydrated cavity at their center (Fig. 3, B and G, and figs. S4C and S6, A to C), they also contain charge-paired interactions (Figs. 3, B and F, and 4, B to E, and figs. S4C and S6, A to C) and bury some hydrophobic residues (Fig. 3, B and G, and fig. S4, B to D). If polar interactions facilitate meaningful, on-pathway contacts, then one might predict that salt-mediated screening of charges would influence the lability of fibrils and perhaps even the rates of fibril formation. To test this prediction, we performed salt gradient experiments (figs. S8A and S9, A and B). We unexpectedly found that 1 M NaCl, which screens charge-charge interactions in proteins, significantly increased the conformational stability of wild-type PrP fibrils (*P* = 0.00035; fig. S8D) but did not significantly influence the lability of E196K fibrils (*P* = 0.0058; fig. S8D). The lag time of E196K fibril formation in the presence of 1 M NaCl is 7.46 ± 1.41 hours (fig. S9D), which is significantly higher than that in the absence of salt (2.00 ± 0.68 hours, *P* = 0.0038; fig. S9D), but the lag time of fibril formation of wild-type PrP in the presence of 1 M NaCl is 5.88 ± 1.07 hours (fig. S9C), which is not significantly higher than that in the absence of salt (2.96 ± 0.38 hours, *P* = 0.011; fig. S9C), suggesting that 1 M salt significantly slowed down fibril formation of E196K but not wild-type PrP. Collectively, these results reveal that high salt concentration differently influences the lability of wild-type and E196K fibrils and the fibrillization kinetics of wild-type and E196K PrPs. It is possible that the differences observed in the melting assay (fig. S7, C to E) are more reflective of the type of interactions holding the cores of the wild-type and E196K fibrils, hydrophobic versus ionic, respectively. The polar contacts holding the E196K fibril together (Figs. 3, B and F, and 4, B to E, and figs. S4C and S6) are intriguing and could potentially inform on the types of structural differences across in vivo structures formed by wild-type or sequence variant PrPs.

## DISCUSSION

We compared the structures of PrP^C^, the E196K fibril, the wild-type PrP fibril, and the 263K prion fibril (Fig. 5). Notably, the PrP molecule adopts largely distinctive secondary structures in four different PrP structures, highlighting the high structural polymorphs and diversity of PrP in soluble and fibrillar forms. The human PrP^C^ contains three α helices, two very short antiparallel β sheets, and a single disulfide bond between Cys^179^ in α2 and Cys^214^ in α3 (*5*, *10*, *21*) (Fig. 5A). Once it folds into its fibrillar form, the PrP subunit undergoes a totally conformational rearrangement. The wild-type human PrP fibril core contains six short β sheets (β1 to β6), a very short interface comprising only three residues 194 to 196, and one disulfide bond between Cys^179^ in a loop (linking β1 to β2) and Cys^214^ in β5 (*33*) (Fig. 5, A and B). In contrast, the E196K fibril core contains a long β sheet (β1), four short β sheets (β2-β5), a long interface comprising residues 194 to 208, and one disulfide bond between Cys^179^ in β1 and Cys^214^ in β5 (Fig. 5, A and C), with a higher β sheet content (77%) than in the wild-type fibril core (55%) (Fig. 5, A to C). The E196K mutation disrupts important interactions in PrP fibrils and results in a rearrangement of the overall structure, forming an amyloid fibril with a dimer interface and a conformation distinct from the wild-type fibril (Fig. 5, B to D). The hamster brain–derived prion (263K prion) fibril core consists of a single protofilament, which contains 11 short β strands (β1 to β11) and one disulfide bond between Cys^179^ in a loop (linking β7 to β8) and Cys^214^ in a long loop (linking β10 to β11) (Fig. 5, A and E) (*34*). The structure of the 263K prion fibril (*34*) is partly compatible with the structures of the wild-type PrP fibril (*33*) and the E196K fibril (this work), where β1, β2, β3, and β6 in the wild-type PrP fibril and β3 in the E196K fibril would correspond to β7, β8, β9, β11, and β10 in the 263K prion fibril core, respectively; in all three models, the fibril features a parallel in-register intermolecular β sheet architecture (Fig. 5, A to C, and E). The three different fibril structures revealed the following key structural differences among the 263K prion fibril, the E196K fibril, and the wild-type fibril. First, the signature of this infectious prion is much higher resistance to proteolytic digestion and substantially longer proteinase K–resistant core compared with PrP fibrils formed in vitro (starting at residue ~95 in contrast to 175 for the E196K fibril and 170 for the wild-type fibril) (Fig. 5, B to E). Second, the number of protofilaments is one for the 263K prion fibril but two for both the E196K fibril and the wild-type PrP fibril (Fig. 5, B to G). Third, the overall folding motif even within the overlapping C-terminal segment of the core of the 263K prion fibril is different from those of E196K and wild-type fibrils. The disulfide β arch in 263K prion contains two N-linked glycans (*34*), but our recombinant PrP fibrils are nonglycosylated [(*33*), this work]. The flanks of the disulfide β arches in the 263K prion fibril (*34*) and the E196K fibril are straighter than that in the wild-type PrP fibril [Fig. 5, B to E, and Extended Data figure 6 in (*34*)]. Fourth, both the 263K prion fibril core (*34*) and the E196K fibril core contain two hydrophilic cavities (Fig. 3, F and G), but the wild-type PrP fibril core contains two hydrophobic cavities (*33*). Last, most of the antibody epitopes and proteolytic cleavage sites lie on the 263K prion fibril core structure [Extended Data figure 7 in (*34*)] but do not lie on core structures of E196K and wild-type fibrils. These results provide structural evidence that different prion strains have distinct conformations, which may underscore a pivotal role of the specific structural features in driving the disease phenotype. The question of what distinguishes infectious ex vivo fibrils from recombinantly generated forms remains an important and open one.

Conformational stabilities of PrP^Sc^ and PrP fibrils assessed by measuring their resistance to chemical and/or thermal denaturation are used to probe the structural differences between prion strains (*13*, *16*, *19*, *20*, *47*, *48*). However, the structural basis of the stability differences between different strains remains to be established (*19*, *20*, *47*, *48*). We previously determined a high-resolution cryo-EM structure of an amyloid fibril formed by full-length wild-type human PrP (*33*). The present study expands these studies, reporting the cryo-EM structure of an amyloid fibril generated from human PrP with the E196K mutation (which corresponds to one of the mutations associated with genetic prion diseases). In that sense, this follow-up study probes the impact of disease-associated sequence variants on the original PrP amyloid fibril structure. In this case, the charge-flipping mutation of Glu to Lys at position 196 disrupts key interactions in the wild-type fibril structure and induces a rearrangement of its core. Our structure data revealed the key structural differences between E196K and wild-type fibrils, including (i) hydrophilic cavity versus hydrophobic cavity; (ii) a mixed hydrophilic and hydrophobic protofibril interface versus a short hydrophilic protofibril interface (the nature of the interface between two protofilaments); and (iii) five versus six β strands in the core. This may help to understand how a single point mutation may lead to formation of a novel structure of a prion fibril with largely distinct properties. It should be mentioned that prion strains are defined by bona fide infectious isolates (which may nonetheless result from different structures) and that a single point mutation may lead to formation of a new prion strain with largely distinct properties (*13*–*20*).

Guest and colleagues reported that three weak salt bridges formed by His^187^ and Asp^202^, Glu^196^ and Asp^202^, and Arg^164^ and Asp^178^, with a distance of 8.0, 7.9, and 6.0 Å, respectively, and a strong salt bridge formed by Arg^208^ and Glu^211^ with a distance of 2.6 Å in the human PrP^C^ were important according to the results of analysis of theoretical mathematics or model mathematics with nuclear magnetic resonance (*49*). Here, a strong salt bridge is defined as an interaction between two groups of opposite charges in which at least one pair of heavy atoms is within distance less than 4 Å (*50*). We previously reported that two pairs of amino acids (Lys^194^ and Glu^196^ and Glu^196^ and Lys^194^) from opposing subunits of the wild-type human PrP fibril formed two strong intermolecular salt bridges, with a distance of 2.2 and 3.1 Å, respectively, to stabilize the short hydrophilic protofibril interface (*33*). We also reported that for the wild-type fibril, two pairs of strong salt bridges formed by Glu^211^ and Arg^228^ from the same subunit, with a distance of 3.2 Å; two pairs of salt bridges formed by Asp^202^ and Lys^204^ from the same subunit, with a distance of 4.3 Å; and eight pairs of weak salt bridges formed by Lys^204^ and Glu^207^, Glu^207^ and Arg^208^, Arg^208^ and Glu^211^, and Arg^220^ and Glu^211^ also from the same subunit, with a distance of 5.0, 5.9, 7.0, and 5.3 Å, respectively, were important according to a high-resolution cryo-EM structure (*33*). Here, we reported a new cryo-EM structure of a PrP amyloid fibril, whose sequence encodes a disease-related sequence variant: E196K. Four pairs of amino acids (Lys^194^ and Glu^207^; Lys^196^ and Glu^200^; Glu^200^ and Lys^196^; and Glu^207^ and Lys^194^) from opposing subunits of the E196K fibril formed four strong intermolecular salt bridges, with a distance of 3.1, 3.4, 3.4, and 3.0 Å, respectively, and Glu^207^ and Arg^208^ from the same subunit of the E196K fibril formed two weak salt bridges, with a distance of 6.3 Å, to stabilize the mixed hydrophilic and hydrophobic protofibril interface. Lys^185^ and Asp^202^ from the same subunit of the E196K fibril formed two strong salt bridges, and His^187^ and Asp^202^ from the adjacent subunit of the E196K fibril formed two salt bridges to stabilize the two hydrophilic cavities.

Asn^181^ and Asn^197^ were previously reported to be glycosylated in wild-type PrP^Sc^ (*24*, *27*, *30*). The recombinant PrPs we used are nonglycosylated. However, in our wild-type PrP fibril model, the side chains of the two Asn residues appear well exposed to solvent (*33*) and thus are able to sterically accommodate bulky, N-linked glycans, as shown by recent molecular dynamics simulations (*51*, *52*). Intriguingly, in the E196K fibril structure, the side chains of Asn^181^ and Asn^197^ are buried in the interior of a hydrophilic core (Fig. 3, A and B). Notably, the side chain of Asn^181^ in the E196K fibril fold appears well exposed to an open area of the hydrophilic core (Fig. 3, A and B) and should thus be able to accommodate a bulky, N-linked glycan. The side chain of Asn^197^, however, appears exposed to a quite crowded area of the hydrophilic core (Fig. 3, A and B) and probably does not have enough space to accommodate a bulky, N-linked glycan. Thus, our E196K fibril model would represent the fibril core of monoglycosylated PrP^Sc^. Very recently, mono-glycosylated PrP^Sc^ has been found to have structural instability (*53*) and form fibrillar plaques (*54*) in some PrP mutations, including genetic CJD-related mutations V180I and T183A (*54*).

In summary, genetic prion disease–related mutation E196K displays a novel amyloid fibril structure revealed by cryo-EM and has a significantly lower conformational stability and protease resistance activity compared to the wild-type fibril. The reported cryo-EM structure of the E196K PrP fibril reveals an unusual overall structure when compared to the wild-type fibril, characterized by a disruption of key salt bridges, a hydrophilic cavity, two unidentified densities flanking the protofibrils, and five instead of six β strands in the core. The structure provides structural evidence for different prion strains and may inspire future research on the mechanism how the familial mutants of PrP can drive disease, given the issue of structural diversity among amyloid fibrils.

## MATERIALS AND METHODS

### Protein expression and purification

A plasmid-encoding, full-length human PrP (23-231) was a gift from G.-F. Xiao (Wuhan Institute of Virology, Chinese Academy of Sciences). The gene for PrP 23-231 was constructed in the vector pET-30a (+), and a PrP mutant E196K was constructed by site-directed mutagenesis using a wild-type PrP template; the primers are shown in table S1. All PrP plasmids were transformed into *E. coli*. Recombinant full-length wild-type human PrP and its variant E196K were expressed from the vector pET-30a (+) in *E. coli* BL21 (DE3) cells (Novagen, Merck, Darmstadt, Germany). PrP proteins were purified by high-performance liquid chromatography on a C4 reverse-phase column (Shimadzu, Kyoto, Japan) as described by Bocharova *et al.* (*41*) and Zhou *et al.* (*39*). After purification, recombinant wild-type PrP^C^ and E196K PrP^C^ were dialyzed against 20 mM tris-HCl buffer (pH 7.4) three times, concentrated, filtered, and stored at −80°C. SDS-PAGE and mass spectrometry were used to confirm that the purified human PrP proteins were single species with an intact disulfide bond. We used a NanoDrop OneC Microvolume UV-Vis Spectrophotometer (Thermo Fisher Scientific) to determine the concentrations of wild-type human PrP^C^ and E196K PrP^C^, using their absorbances at 280 nm and the molar extinction coefficients calculated from the composition of the proteins (http://web.expasy.org/protparam/).

### PrP fibril formation

Full-length recombinant wild-type human PrP^C^ and E196K PrP^C^ (40 μM) were incubated in 20 mM tris-HCl buffer (pH 7.4) containing 2 M guanidine hydrochloride (GdnHCl) with shaking at 180 rpm at 37°C for 9 to 11 and 7 to 9 hours, respectively, and the wild-type and E196K fibrils were collected. Large aggregates in E196K fibril samples and the wild-type fibril samples were removed by centrifugation for 5000*g* at 4°C for 10 min. The supernatants were then dialyzed against 20 mM NaAc buffer (pH 5.0) three times to ensure that GdnHCl had been removed. After dialysis, E196K fibril samples and the wild-type fibril samples were purified by ultracentrifugation for 100,000*g* for 30 min twice and washed with 20 mM NaAc buffer (pH 5.0). The pellets containing E196K fibrils or the wild-type fibrils were resuspended in 100 μl of 20 mM NaAc buffer (pH 5.0) and not treated with proteinase K. We used a NanoDrop OneC Microvolume UV-Vis Spectrophotometer (Thermo Fisher Scientific) to determine the concentrations of the E196K fibril and the wild-type PrP fibril using their absorbances at 280 nm and the molar extinction coefficients calculated from the composition of PrPs (http://web.expasy.org/protparam/).

### Congo red binding assays

E196K fibrils were analyzed by Congo red binding assays. A stock solution of 200 μM Congo red was prepared in phosphate-buffered saline and filtered through a filter of 0.22-μm pore size before use. In a typical assay, the E196K fibril sample was mixed with a solution of Congo red to yield a final Congo red concentration of 50 μM and a final PrP concentration of 10 μM, and the absorbance spectrum between 400 and 700 nm was then recorded on a Cytation 3 Cell Imaging Multi-Mode Reader (BioTek).

### TEM of E196K fibrils

E196K fibrils were examined by TEM of negatively stained samples. Ten microliters of E196K fibril samples (~13 μM) were loaded on copper grids for 30 s and washed with H_2_O for 10 s. Samples on grids were then stained with 2% (w/v) uranyl acetate for 30 s and dried in air at 25°C. The stained samples were examined using a JEM-1400 Plus transmission electron microscope (JEOL) operating at 100 kV.

### Proteinase K digestion assay

E196K fibrils were assessed by proteinase K digestion. The E196K fibril samples were incubated with proteinase K at a protease:PrP molar ratio of 1:500 to 1:100 for 1 hour at 37°C. Digestion was stopped by the addition of 2 mM phenylmethylsulfonyl fluoride, and the samples were analyzed in 15% SDS-PAGE and detected by silver staining.

### Cryo-EM of PrP fibrils

E196K fibrils were produced as described above. An aliquot of 3.5 μl of ~13 μM E196K fibril solution was applied to glow-discharged holey carbon grids (Quantifoil Cu R1.2/1.3, 300 mesh), blotted for 3.5 s and plunge-frozen in liquid ethane using an FEI Vitrobot Mark IV. The grids were examined using an FEI Talos F200C microscope, operated at 200 kV, and equipped with a field emission gun and an FEI Ceta camera (Thermo Fisher Scientific). The cryo-EM micrographs were acquired on an FEI Titan Krios microscope operated at 300 kV (Thermo Fisher Scientific) and equipped with a Gatan K2 Summit camera. A total of 4883 movies were collected in counting mode at a nominal magnification of ×29,000 (pixel size, 1.014 Å) and a dose of 8 e^−^ Å^−2^ s^−1^ (see Table 1). An exposure time of 8 s was used, and the resulting videos were dose-fractionated into 40 frames. A defocus range of −1.5 to −3.0 μm was used.

### Helical reconstruction

All 40 video frames were aligned, summed, and dose-weighted by MotionCor2 and further binned to a pixel size of 1.014 Å (*55*). Contrast transfer function estimation of aligned, dose-weighted micrographs was performed by CTFFIND4.1.8 (*56*). Subsequent image-processing steps, which include manual picking, particle extraction, 2D and 3D classifications, 3D refinement, and postprocessing, were performed by RELION3.1 (*43*).

In total, 25,301 fibrils were picked manually from 4883 micrographs, and 686- and 400-pixel boxes were used to extract particles by 90% overlap scheme. Two-dimensional classification of 686-box size particles was used to calculate the initial twist angle. In regard to helical rise, 4.8 Å was used as the initial value. Particles were extracted into 400-box sizes for further processing. After several iterations of 2D and 3D classifications, particles with the same morphology were picked out. Local searches of symmetry in 3D classification were used to determine the final twist angle and rise value. The 3D initial model was built by selected 2D classes; 3D classification was performed several times to generate a proper reference map for 3D refinement. Three-dimensional refinement of the selected 3D classes with appropriate reference was performed to obtain final reconstruction. The final map of E196K fibrils was convergent with a rise of 2.41 Å and a twist angle of 179.66°. Postprocessing was preformed to sharpen the map with a *B* factor of −43.26 Å^2^. On the basis of the gold standard Fourier shell correlation (FSC) = 0.143 criteria, the overall resolution was reported as 3.07 Å. The statistics of cryo-EM data collection and refinement is shown in Table 1.

### Atomic model building and refinement

COOT (*57*) was used to build and modify the atomic model of the E196K fibril on the basis of the cryo-EM structure of the wild-type PrP fibril [Protein Data Bank (PDB) 6LNI] (*33*). The model with three adjacent layers was generated for structure refinement. The model was refined using the real-space refinement program in PHENIX (*58*).

### Global denaturation of E196K and wild-type fibrils analyzed by ThT fluorescence and SDS-PAGE

Amyloid fibrils were produced from wild-type and E196K PrPs incubated in 20 mM tris-HCl buffer (pH 7.4) containing 2 M guanidine hydrochloride and shaking at 37°C for 10 hours. Samples (40 μM) of the PrP fibrils were incubated for 1 hour at 25°C in the presence of different concentrations of guanidine thiocyanate (GdnSCN) and 0, 150 mM, or 1 M NaCl. The concentration of GdnSCN was then adjusted to 0.35 M, followed by a thioflavin T (ThT) binding assay. A Cytation 3 Cell Imaging Multi-Mode Reader (BioTek) was used to the ThT fluorescence produced, with excitation at 450 nm and emission at 480 nm. The half-concentration at which the ThT fluorescence intensity of PrP fibrils is decreased by 50% (*C*_1/2_) of the E196K fibril and the wild-type fibril was determined using a sigmoidal equation (*59*, *60*) using the above ThT fluorescence data. Statistical analyses were performed using the revised Student’s *t* test. Values of *P* < 0.005 indicate statistically significant differences. The following notation is used throughout: *P < 0.005, **P < 0.001, and ***P < 0.0001 relative to control. Samples (40 μM) of the PrP fibrils were also dialyzed against 20 mM NaAc buffer (pH 5.0) for three times and diluted to a final concentration of 16 μM using 10% SDS. The final concentration SDS was 6%. The PrP fibrils were incubated with 6% SDS for 5 min under a thermal gradient from 25 to 100°C, mixed with the 5× loading buffer (without SDS, β-mercaptoethanol, and heating) and separated by 12.5% SDS-PAGE. The soluble PrP monomers were detected by SDS-PAGE with Coomassie Blue R250 staining. The normalized amount of soluble PrP monomers was determined as a ratio of the density of soluble PrP monomer band at each temperature over the density of soluble PrP monomer band at 100°C.

### Fibrillization kinetics of wild-type PrP and its variant E196K analyzed by ThT fluorescence

Samples (20 μM) of wild-type and E196K PrPs were incubated in 20 mM tris-HCl buffer (pH 7.4) containing 2 M guanidine hydrochloride and shaking at 180 rpm and 37°C for 13 hours in the absence or presence of 50, 150, 300, and 500 mM or 1 M NaCl, and analyzed by a ThT binding assay. The final concentrations of PrP and ThT were 1 and 125 μM, respectively. A Cytation 3 Cell Imaging Multi-Mode Reader (BioTek) was used to the ThT fluorescence produced, with excitation at 450 nm and emission at 480 nm. The lag times of fibril formation of wild-type and E196K PrPs in the presence of different concentrations of salt were determined using a sigmoidal equation (*59*, *60*) using the above ThT fluorescence data. Statistical analyses were performed using the revised Student’s *t* test. Values of *P* < 0.005 indicate statistically significant differences. The following notation is used throughout: *P < 0.005, **P < 0.001, and ***P < 0.0001 relative to control.

## Notes

View/request a protocol for this paper from *Bio-protocol*.
