# Supplementary material for: Genetic prion disease–related mutation E196K displays a novel amyloid fibril structure revealed by cryo-EM
Source: Sci Adv. 2021 Sep 8;7(37):eabg9676. doi: 10.1126/sciadv.abg9676 (PMC8442898; doi:10.1126/sciadv.abg9676)
Supplement: Supplementary file 1 — Table S1 Figs. S1 to S9 [file sciadv.abg9676_sm.pdf]

Science Advances
AAAS

Supplementary Materials for

Genetic prion disease–related mutation E196K displays a novel amyloid fibril structure revealed by cryo-EM

Li-Qiang Wang, Kun Zhao, Han-Ye Yuan, Xiang-Ning Li, Hai-Bin Dang, Yeyang Ma, Qiang Wang, Chen Wang, Yunpeng Sun, Jie Chen, Dan Li, Delin Zhang, Ping Yin, Cong Liu*, Yi Liang*

*Corresponding author. Email: liulab@sioc.ac.cn (C.L.); liangyi@whu.edu.cn (Y.L.)

Published 8 September 2021, *Sci. Adv.* **7**, eabg9676 (2021)
DOI: 10.1126/sciadv.abg9676

This PDF file includes:

Table S1
Figs. S1 to S9

Supplementary Materials

table S1.

The primers designed for full-length human PrP with E196K mutation

S-E196K	5’CACCAAGGGGAAGAACTTCACCGAG3’
A-E196K	5’GTGAAGTTCTTCCCCTTGGTGGTTGTG3’

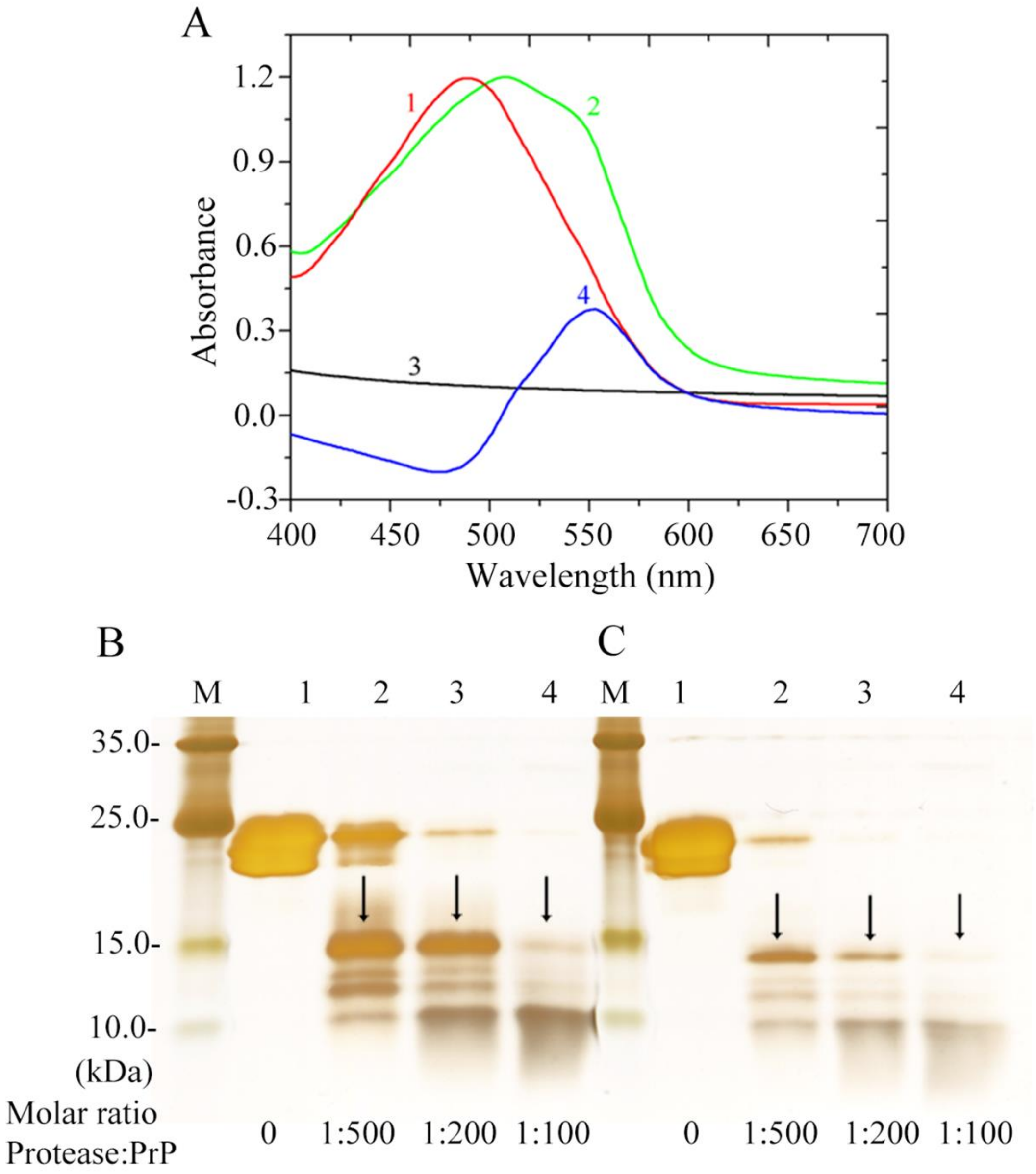

figure S1.

E196K fibrils bind to Congo red and are proteinase K resistant. (**A**) Amyloid fibrils of full-length human PrP with E196K mutation analyzed by Congo red binding assays. The difference spectra (Curve 4, blue) with the maximum absorbance at 550 nm were obtained by subtracting the absorbance spectra of E196K fibrils alone (Curve 3, black) and Congo red alone (Curve 1, red) with the maximum absorbance at 490 nm from those of E196K fibrils + Congo red (Curve 2, green). Congo red binding assays were carried out at 37°C. (**B** and **C)** Amyloid fibrils of full-length wild-type human PrP (B) and its variant E196K (C) analyzed by concentration-dependent proteinase K digestion assays (*33*, *39*). Protease-resistant core fragments of 15- to 16-kDa (B) and 14- to 15-

kDa (C) are highlighted using black arrows. Samples were treated with proteinase K for 1 h at 37°C at protease:PrP molar ratios of 1:500 (lane 2), 1:200 (lane 3), and 1:100 (lane 4). The control with no protease was loaded in lane 1. Molecular weight markers were loaded on lane M. Protein fragments were separated by SDS-PAGE and detected by silver staining. These experiments were repeated three times with different batches of fibrils and similar results.

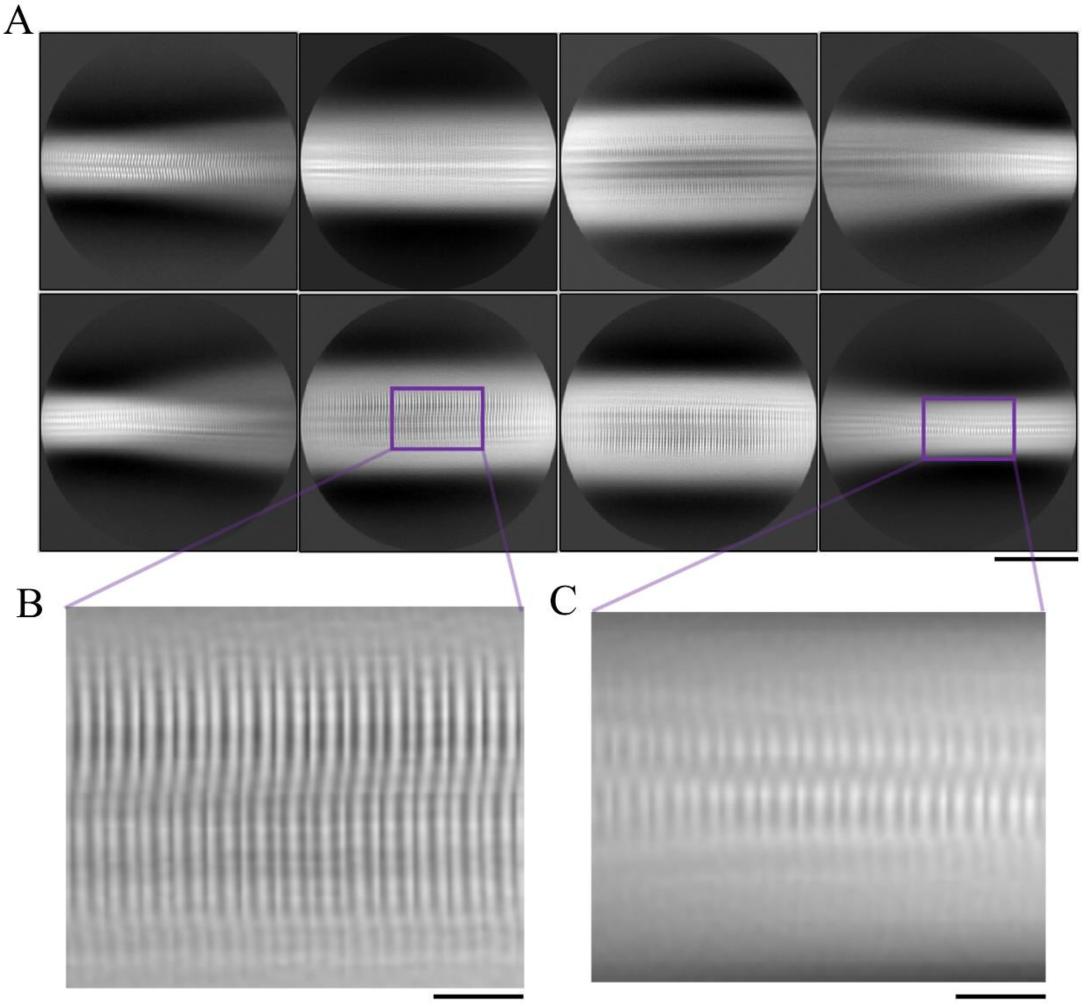

figure S2.

Cryo-EM images of E196K fibrils. (**A**) Reference-free 2D class averages of E196K fibrils showing two protofibrils intertwined. Scale bar, 10 nm. (**B** and **C)** Enlarged images of (A) showing two protofibrils arranged in a staggered manner. Scale bar, 2 nm.

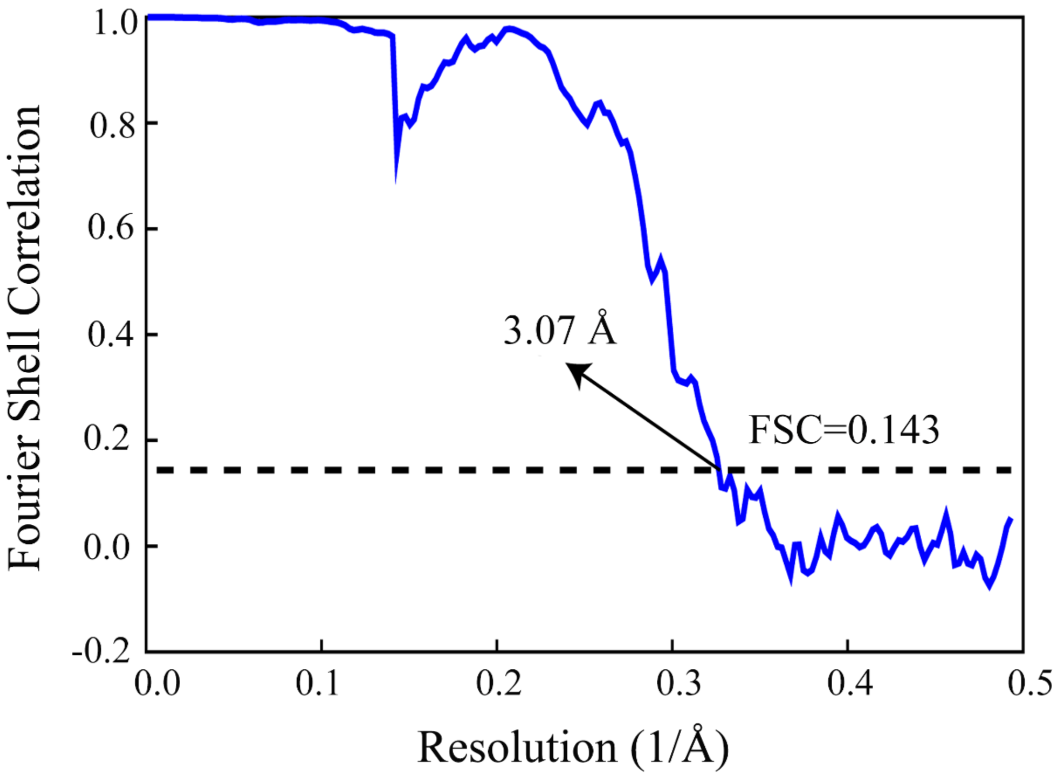

figure S3.

Global resolution estimate for the E196K fibril reconstructions. Gold-standard refinement was used for estimation of the density map resolution. The global resolution of 3.07 Å was calculated using a Fourier shell correlation (FSC) curve cut-off at 0.143.

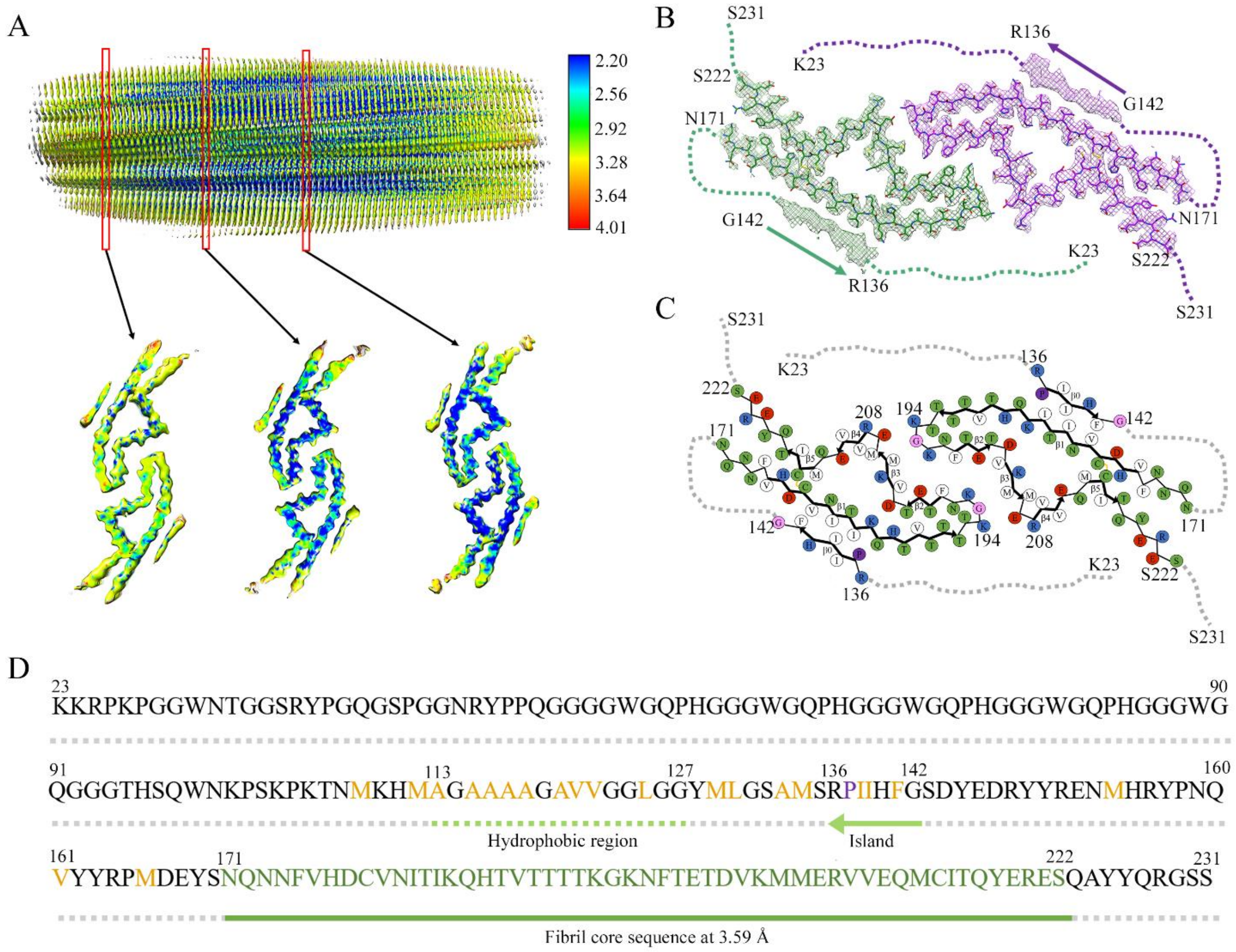

figure S4.

Cryo-EM structure of E196K fibrils at 3.59 Å. (**A**) Local resolution estimate for the E196K fibril reconstructions at 3.59 Å. The unmasked density map of E196K fibrils is colored according to local resolution estimated by ResMap. The three enlarged cross sections show the left top view of the density map of two protofibrils, and each monomer has an island. The color key on the right shows the local structural resolution in angstroms (Å) and the colored map indicates the local resolution ranging from 2.2 to 4.01 Å. (**B**) Cryo-EM map of E196K fibrils at 3.59 Å with a fibril core comprising residues 171 to 222 from E196K. Two protofibrils with two islands (marked by arrow) are colored green and purple, respectively. Each island, probably comprising residues

136 to 142 from E196K and forming a β strand (β0), is located on the opposing side of hydrophobic side chains of Val^{180}, Ile^{182}, and Ile^{184} in each monomer. The side chains of Val^{180}, Ile^{182}, Ile^{184}, Pro^{137}, Ile^{139}, and Phe^{141} forms a hydrophobic steric zipper-like interface, thereby stabilizing the E196K fibrils. (**C**) Schematic view of the E196K fibril core at 3.59 Å. Residues are colored as follows: white, hydrophobic; green, polar; red and blue, negatively and positively charged, respectively; purple, proline; and magenta, glycine. Six β strands (β0 to β5) are indicated with bold lines. E196K fibrils are stabilized by a disulfide bond (yellow line) formed between Cys^{179} and Cys^{214} in each monomer, also visible in (B). (**D**) The primary sequence of E196K, in which the green bar marks the E196K fibril core comprising residues 171 to 222 at 3.59 Å. The green arrow marks an island flanking each protofibril, and the green dotted line marks a hydrophobic region of human PrP^C. Residues are colored as follows: orange, hydrophobic; and green, the E196K fibril core at 3.59 Å. The dotted lines correspond to residues 23 to 135/ 143 to 170/ 223 to 231 not modeled in the cryo-EM density.

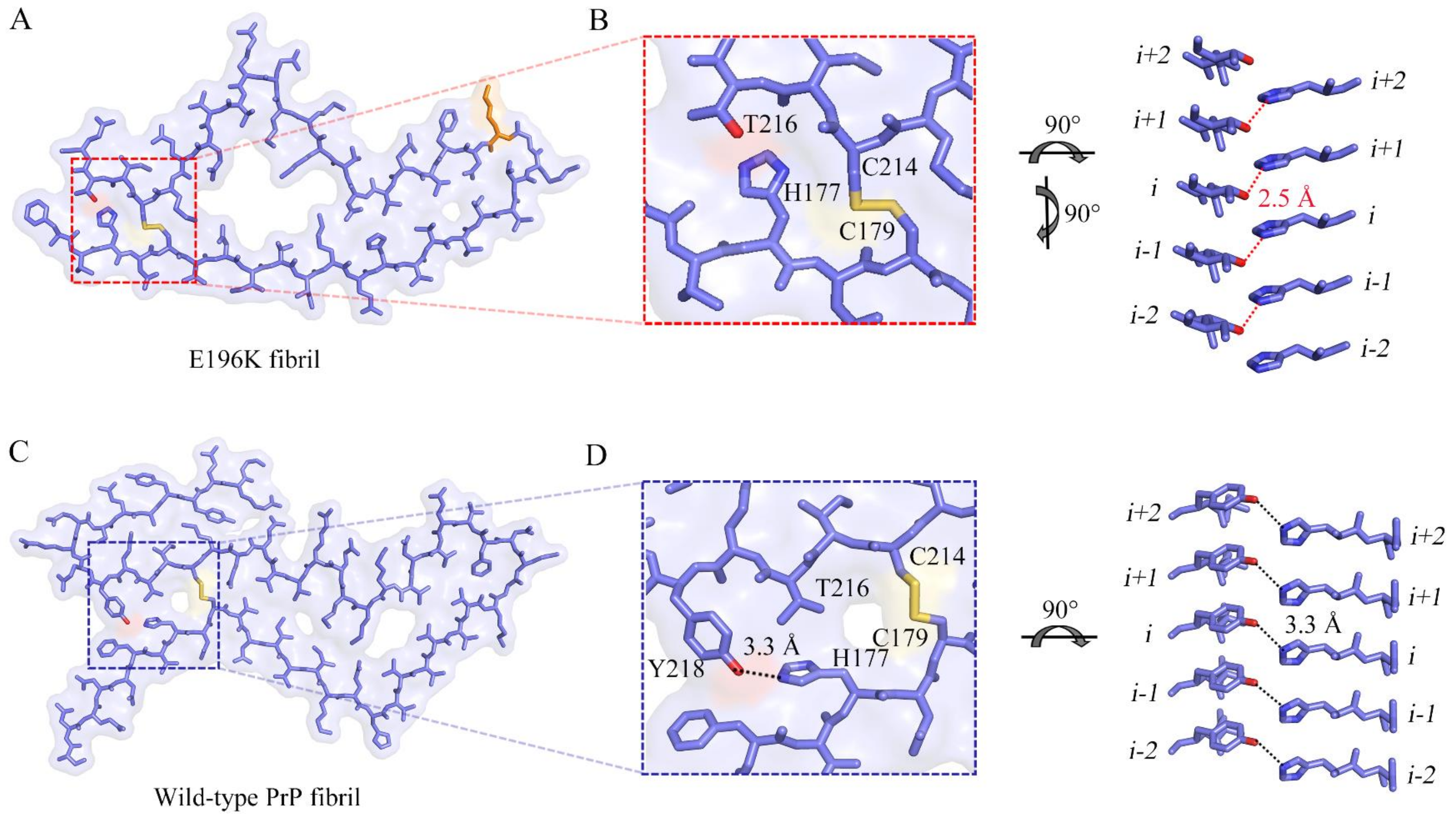

figure S5.

Close-up view of the stick representation of the structures of E196K and wild-type fibrils. (**A**) A space-filled model overlaid onto a stick representation of the E196K fibril in which one protofibril is shown in blue. An E196K protofibril is stabilized by one disulfide bond (yellow line)

between Cys^{179} and Cys^{214}, and the disulfide bond region is magnified in (B). Lys196 in E196K variant is highlighted in orange. (**B**) A magnified top view of the disulfide bond region of an E196K protofibril, where a disulfide bond (yellow line) is formed between Cys^{179} and Cys^{214} and a hydrogen bond is formed between His^{177} and Thr^{216}. A side view (right) highlighting a hydrogen bond between His^{177} (*i*) and Thr^{216} from its opposing adjacent subunit (*i–1*), with a distance of 2.5 Å (red). (**C**) A space-filled model overlaid onto a stick representation of the wild-type PrP fibril (PDB 6LNI) (*33*) in which one protofibril is shown in blue. A wild-type PrP protofibril is also stabilized by one disulfide bond (yellow line) between Cys^{179} and Cys^{214}, and the disulfide bond region is magnified in (D). (**D**) A magnified top view of the disulfide bond region of a wild-type PrP protofibril, where a disulfide bond (yellow line) is formed between Cys^{179} and Cys^{214} and a hydrogen bond is formed between His^{177} and Tyr^{218}. A side view (right) highlighting a hydrogen bond between His^{177} (*i*) and Tyr^{218} from its opposing subunit (*i*), with a distance of 3.3 Å (black).

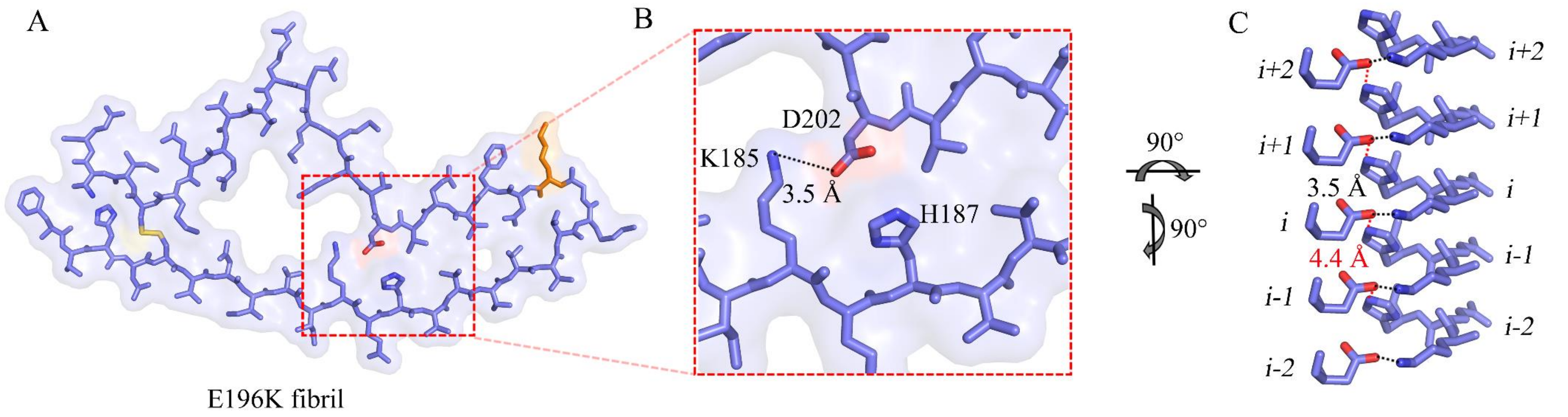

figure S6.

Close-up view of the stick representation of the structure of the E196K fibril. (**A**) A space-filled model overlaid onto a stick representation of the E196K fibril in which one protofibril is shown in blue. An E196K protofibril core is composed of a hydrophilic cavity which is stabilized by two salt bridges, and the salt bridge region is magnified in (B). Lys^{196} in E196K variant is highlighted in orange. **(B)** A magnified top view of the salt bridge region of an E196K protofibril, where two pairs of amino acids (Lys^{185} and Asp^{202}; His^{187} and Asp^{202}) form two salt bridges. **(C)** A side view highlighting a strong salt bridge between Lys^{185} (*i*) and Asp^{202} from the same subunit (*i*), with a distance of 3.5 Å (black), and another salt bridge between His^{187} (*i*) and Asp^{202} from its adjacent subunit (*i*+1), with a distance of 4.4 Å (red).

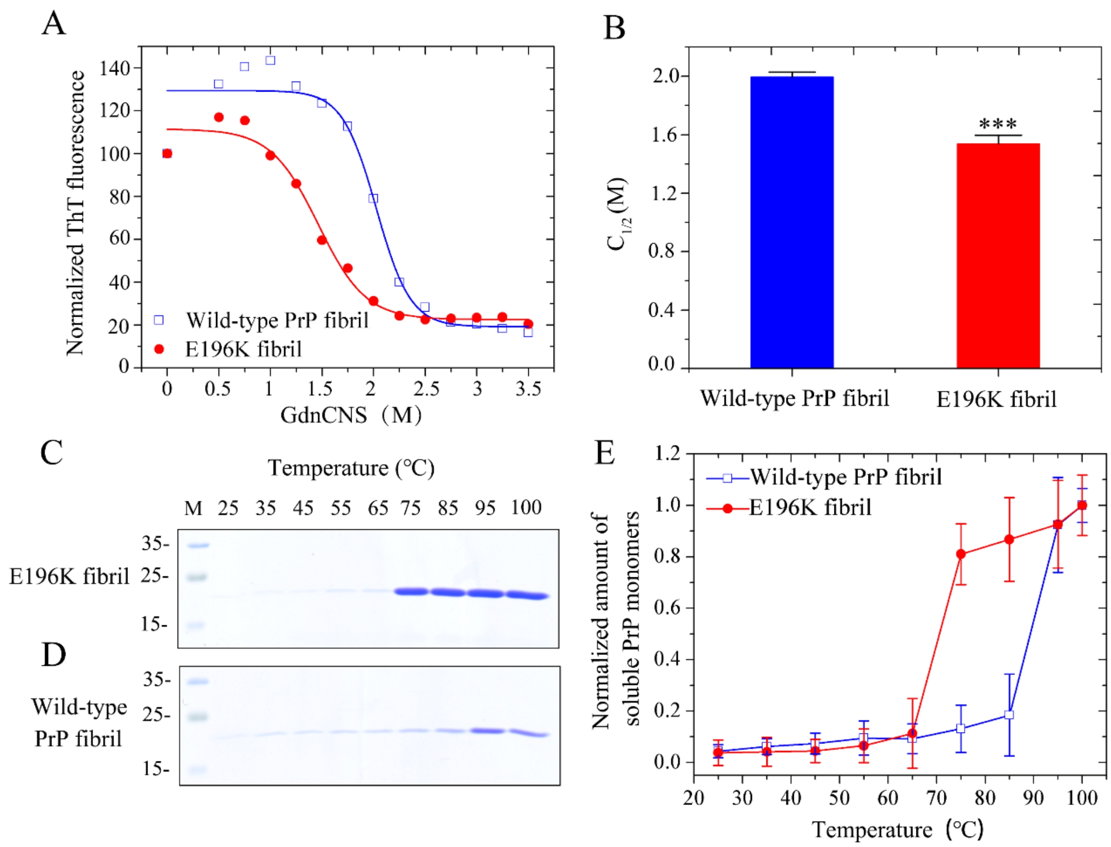

figure S7.

The E196K mutation significantly decreases the conformational stability of PrP fibrils. Amyloid fibrils were produced from wild-type and E196K PrPs incubated in 20 mM tris-HCl buffer (pH 7.4) containing 2 M guanidine hydrochloride and shaking at 37°C for 10 hours. (**A**) GdnSCN-induced denaturation profiles were monitored for the E196K fibril (red) and the wild-type PrP fibril (blue). Samples (40 μM) of the PrP fibrils were incubated for 1 hour at 25°C in the presence of different concentrations of GdnSCN. The concentration of GdnSCN was then adjusted to 0.35 M, followed by a ThT binding assay. The solid lines show the best sigmoidal fit for the ThT intensity-time curves. (**B**) The $C_{1/2}$ (the half-concentration at which the ThT fluorescence intensity of PrP fibrils is decreased by 50%) values of the E196K fibril (red) and the wild-type PrP fibril (blue) were determined using a sigmoidal equation (*59*, *60*) using the ThT fluorescence data obtained, and are expressed as mean ± SD of the values obtained in 4 independent experiments. The E196K fibril, $P = 0.0000094$. The revised Student's t test was used to perform statistical analyses. Values of $P < 0.005$ indicate statistically significant differences. The following notation is used throughout: $*P <$

0.005, $**P < 0.001$, and $***P < 0.0001$ relative to the wild-type PrP fibril (a control). (**C** and **D**) Thermal stabilities of the E196K fibril (C) and the wild-type PrP fibril (D) at different temperatures were determined by SDS-PAGE. Samples (40 μM) of the PrP fibrils were incubated with 6% SDS for 5 min under each temperature indicated, and the soluble PrP monomers were detected by SDS-PAGE with Coomassie Blue R250 staining. Molecular weight markers were loaded on lane M. (**E**) The normalized amount of soluble PrP monomers was determined as a ratio of the density of soluble PrP monomer band at each temperature indicated over the density of soluble PrP monomer band at 100°C, and expressed as the mean ± SD (with error bars) of values obtained in 3 independent experiments. The melting temperature (T_m) values of the E196K fibril (red) and the wild-type PrP fibril (blue) were ~75°C and ~95°C, respectively, at which the band intensity of soluble PrP monomers almost reached a maximum and the normalized amount of soluble PrP monomers was larger than 0.8. The E196K fibril has a significantly lower conformational stability compared to the wild type. These experiments were repeated three times with different batches of fibrils and similar results.

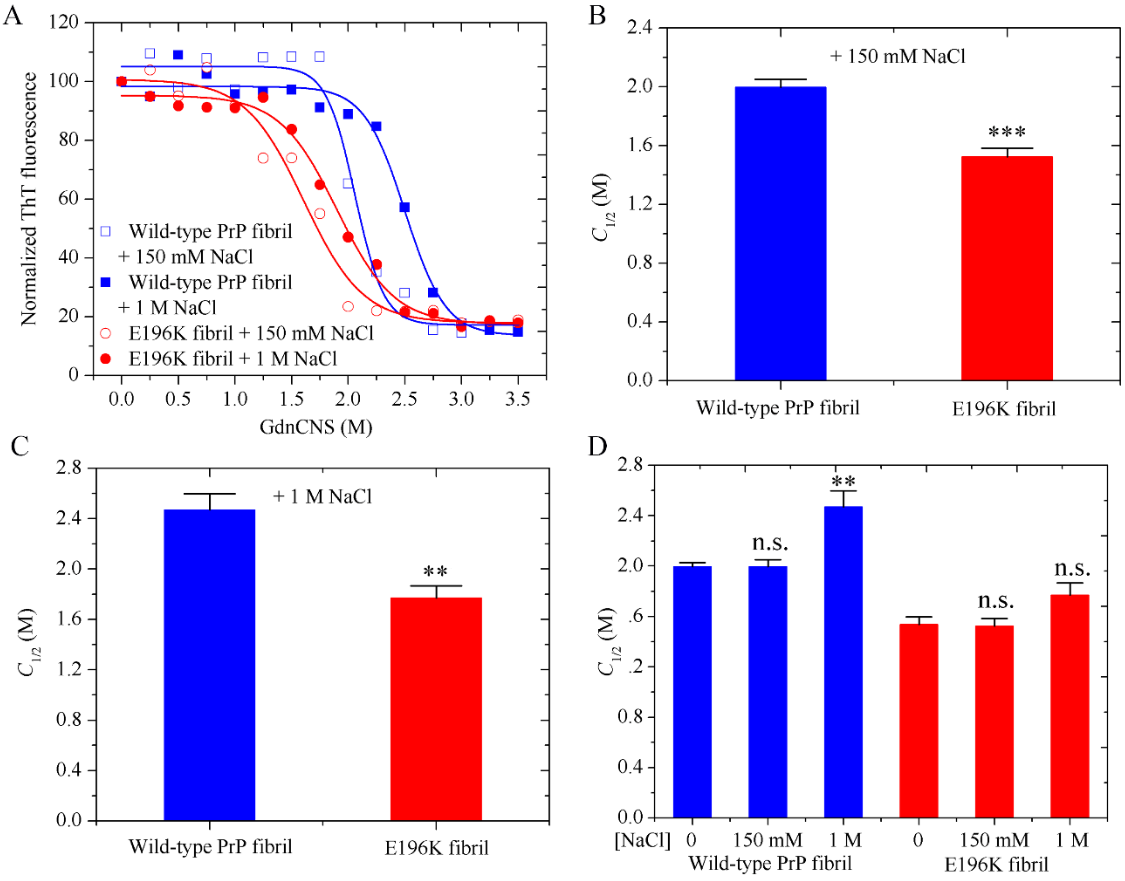

figure S8.

The E196K mutation also significantly decreases the conformational stability of PrP fibrils in the presence of 150 mM or 1 M NaCl. Amyloid fibrils were produced from wild-type and E196K PrPs incubated in 20 mM tris-HCl buffer (pH 7.4) containing 2 M guanidine hydrochloride and shaking at 37°C for 10 hours. (**A**) GdnSCN-induced denaturation profiles were monitored for the E196K fibril (red) and the wild-type PrP fibril (blue). Samples (40 µM) of the PrP fibrils were incubated for 1 hour at 25°C in the presence of different concentrations of GdnSCN and 150 mM (open circle or square) or 1 M NaCl (solid circle or square). The concentration of GdnSCN was then adjusted to 0.35 M, followed by a ThT binding assay. The solid lines show the best sigmoidal fit for the ThT intensity-time curves. (**B** to **D**) The $C_{1/2}$ (the half-concentration at which the ThT fluorescence intensity of PrP fibrils is decreased by 50%) values of the E196K fibril (red) and the wild-type PrP fibril (blue) in the presence of 150 mM (B), 1 M (C) or 0 to 1 M (D) NaCl were determined using a sigmoidal equation (*59*, *60*) using the ThT fluorescence data obtained, and are expressed as mean

± SD of the values obtained in 4 independent experiments. The E196K fibril, P = 0.000022 (B) or 0.00012 (C). The wild-type PrP fibril or the E196K fibril + 150 mM NaCl, P = 0.99 or 0.77 (D); and the wild-type PrP fibril or the E196K fibril + 1 M NaCl, P = 0.00035 or 0.0058 (D). (D) 1 M NaCl significantly increased the conformational stability of the wild-type PrP fibrils but did not significantly influence the lability of the E196K fibrils. The revised Student's t test was used to perform statistical analyses. Values of $P < 0.005$ indicate statistically significant differences. The following notation is used throughout: $*P < 0.005$, $**P < 0.001$, and $***P < 0.0001$ relative to the wild-type PrP fibril (a control) (B and C) or relative to the wild-type PrP fibril (or the E196K fibril) in the absence of salt (a control) (D). n.s., no significance.

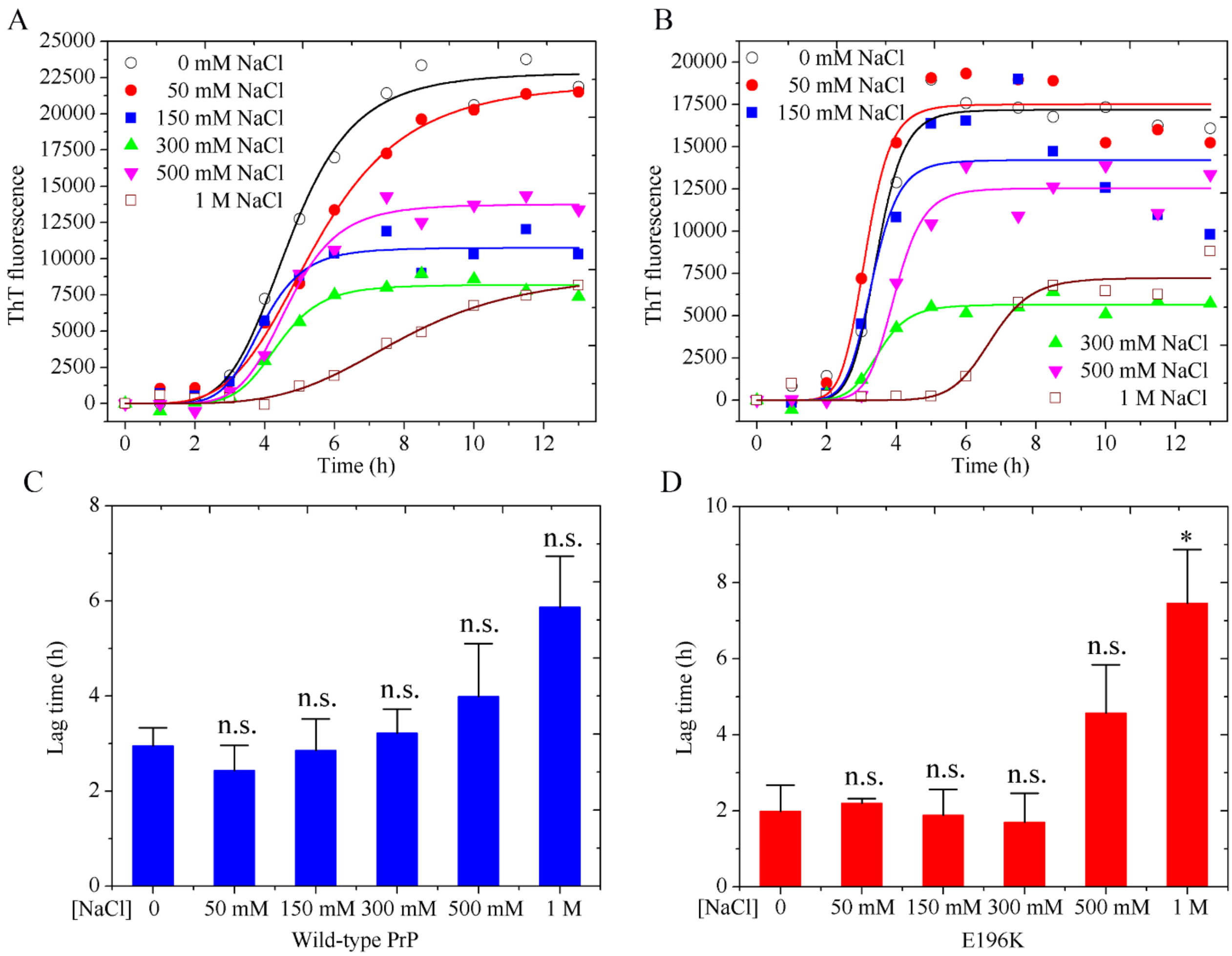

figure S9.

High salt concentration differently influences the fibrillization kinetics of wild-type PrP and its variant E196K. (**A** and **B**) Samples (20 μM) of full-length wild-type human PrP (A) and its variant E196K (B) were incubated in 20 mM tris-HCl buffer (pH 7.4) containing 2 M guanidine hydrochloride and shaking at 37°C for 13 hours, in the absence (black) or presence of 50 (red), 150 (blue), 300 (green), 500 (magenta) mM or 1 M (wine) NaCl, and analyzed by a ThT binding assay. The solid lines show the best sigmoidal fit for the ThT intensity-time curves. (**C** and **D**) The lag times of fibril formation of wild-type PrP (C) and E196K (D) in the presence of different concentrations of salt were determined using a sigmoidal equation (*59*, *60*) using the ThT fluorescence data obtained, and are expressed as mean ± SD of the values obtained in 3 independent experiments. Wild-type PrP or E196K + 50 mM NaCl, $P = 0.24$ (C) or 0.64 (D); wild-type PrP or E196K + 150 mM NaCl, $P = 0.83$ (C) or 0.86 (D); wild-type PrP or E196K + 300 mM NaCl, $P = 0.50$ (C) or 0.64 (D); wild-type PrP or E196K

+ 500 mM NaCl, $P = 0.20$ (C) or 0.036 (D); and wild-type PrP or E196K + 1 M NaCl, $P = 0.011$ (C) or 0.0038 (D). 1 M NaCl significantly slowed down fibril formation of E196K but not wild-type PrP. The revised Student's *t* test was used to perform statistical analyses. Values of $P < 0.005$ indicate statistically significant differences. The following notation is used throughout: $*P < 0.005$, $**P < 0.001$, and $***P < 0.0001$ relative to wild-type PrP or E196K in the absence of salt (a control). n.s., no significance.
